# m6A Modification-Mediated *DUXAP8* Regulation of Malignant Phenotype and Chemotherapy Resistance of Hepatocellular Carcinoma Through miR-584-5p/MAPK1/ERK Pathway Axis

**DOI:** 10.3389/fcell.2021.783385

**Published:** 2021-12-09

**Authors:** Zefeng Liu, Jin Lu, He Fang, Jiyao Sheng, Mengying Cui, Yongsheng Yang, Bo Tang, Xuewen Zhang

**Affiliations:** ^1^ Department of Hepatobiliary Pancreatic Surgery, The Second Hospital of Jilin University, Changchun, China; ^2^ Jilin Engineering Laboratory for Translational Medicine of Hepatobiliary and Pancreatic Diseases, Changchun, China; ^3^ Cancer Center, The First Hospital of Jilin University, Changchun, China; ^4^ Department of Health Sciences, Hiroshima Shudo University, Hiroshima, Japan

**Keywords:** DUXAP8, hepatocellular carcinoma, m6A methylation modification, chemotherapy resistance, malignant phenotype, miR-584-5p, MAPK1

## Abstract

Hepatocellular carcinoma (HCC) has a poor prognosis due to its high malignancy, rapid disease progression, and the presence of chemotherapy resistance. Long-stranded non-coding RNAs (lncRNAs) affect many malignant tumors, including HCC. However, their mechanism of action in HCC remains unclear. This study aimed to clarify the role of *DUXAP8* in regulating the malignant phenotype and chemotherapy resistance in HCC. Using an *in vivo* xenograft tumor model, the regulatory functions and mechanisms of lncRNA *DUXAP8* in the progression and response of HCC to chemotherapy were explored. It was found that *DUXAP8* was significantly upregulated in a patient-derived xenograft tumor model based on sorafenib treatment, which is usually associated with a relatively poor prognosis in patients. In HCC, *DUXAP8* maintained its upregulation in the expression by increasing the stability of m6A methylation-mediated RNA. *DUXAP8* levels were positively correlated with the proliferation, migration, invasion, and chemotherapy resistance of HCC *in vivo* and *in vitro*. In the mechanistic study, it was found that *DUXAP8* competitively binds to miR-584-5p through a competing endogenous RNA (ceRNA) mechanism, thus acting as a molecular sponge for miR-584-5p to regulate *MAPK1* expression, which in turn activates the *MAPK/ERK* pathway. These findings can provide ideas for finding new prognostic indicators and therapeutic targets for patients with HCC.

## Introduction

Hepatocellular carcinoma (HCC) is one of the five most common malignant tumors globally (Zhu et al., 2019). Worldwide, nearly 700,000 people die each year from HCC (Fang et al., 2018; Wu et al., 2019), and its death toll ranks second among all malignant tumor-associated deaths. HCC poses a serious health burden globally (Petrick et al., 2020; Reinders et al., 2020; Yang et al., 2020). In China, the number of deaths due to HCC is the second-highest among all malignant tumors, which come right after lung cancer (Raoul and Edeline, 2020). Despite recent improvements in surgical techniques and chemotherapy regimens, the prognosis of HCC remains poor, and its prevalence is approaching its mortality (Jemal et al., 2011). Particularly, the occurrence of chemotherapy resistance has severely hampered the therapeutic outcome of HCC. Sorafenib, a tyrosine kinase inhibitor, is currently the first-line medication for treating unresectable HCC (Bruix et al., 2019) and can extend the survival of HCC patients by an average of 12.3 months (Kudo et al., 2018; Rimassa and Worns, 2020), but this benefit of increased survival may be lost due to the emergence of drug resistance. The etiology of HCC and the mechanisms of chemotherapy resistance are still unclear; thus, a deeper understanding of genetics and epigenetic mechanisms behind the development and progression of HCC and chemotherapy resistance is required to develop more effective therapeutic intervention strategies.

The development of whole-genome and transcriptome sequencing technologies has revealed the composition of the human genome, where less than 2% of genes are protein-coding genes and the rest are all non-coding genes (Harrow et al., 2012; Shi et al., 2016). There is increasing evidence that complex functions in living organisms may be regulated by a range of RNAs from non-coding regions of the genome. Long non-coding RNAs (LncRNAs) represent a group of RNA molecules with a transcript length exceeding 200 nucleotides and encode only short polypeptides or do not encode proteins (Zheng et al., 2015). Recent studies have indicated that lncRNAs are involved in many critical biological processes, such as X-chromosome inactivation, stem cell maintenance, transcriptional regulation, and epigenetic regulation (Zhang et al., 2018). Additionally, lncRNAs are involved in the regulation of many diseases, especially playing an essential role in the occurrence and development of malignant tumors. Some lncRNAs act as protein co-regulators directly binding to their helper proteins and regulating the expression of downstream tumor-associated genes (Chen et al., 2016; Yuan et al., 2016; Zhu et al., 2016). Another part of lncRNAs act as competitive endogenous RNAs (ceRNAs) (Yuan et al., 2014; Li et al., 2015b), and such lncRNAs achieve microRNA detachment and regulate the expression of microRNA-targeted oncogenes or tumor suppressor genes through molecular sponge effects. These studies suggested that the abnormal expression of lncRNAs plays an essential role in tumor development.

The pseudogene-derived lncRNA, Double Homeobox A Pseudogene 8 (*DUXAP8*), is located on chromosome 22q11.1 with a full length of 2,107 bp (Sun et al., 2017). Recently, *DUXAP8* has been shown to be highly expressed in various malignant tumors. It has been shown that *DUXAP8* can significantly inhibit the expression of *PLEKHO1* in gastric cancer, enhancing the proliferation and migration of tumor cells (Ma et al., 2017a). In glioma, downregulation of *DUXAP8* inhibits the proliferation of tumor cells (Zhao et al., 2019). In non-small cell lung cancer (NSCLC), *DUXAP8* promotes tumor cell proliferation and invasion through epigenetic silencing of *Egr1* and *RHOB* (Sun et al., 2017). In pancreatic cancer, *DUXAP8* promotes tumor growth through epigenetic silencing of *CDKN1A* and *KLF2* (Lian et al., 2018). While the mechanism of action of *DUXAP8* in HCC is unclear, Wang et al. (2020) reported that *DUXAP8* can be used for the diagnosis of HCC and can predict the prognosis of HCC. In this study, it was found that *DUXAP8* was highly expressed in liver cancer tissues and cells, where high expression of *DUXAP8* often predicts poor prognosis in patients. In *in vivo* and *in vitro* experiments, it was found that overexpression of *DUXAP8* promoted the malignant phenotype and chemotherapy resistance in HCC. Mechanistically, it was found that *DUXAP8* upregulated *MAPK1* through competitive binding to miR-584-5p, which activated the *MAPK/ERK* pathway and promoted the proliferation, invasive migration, stemness maintenance, and chemotherapy resistance of HCC. These results provided a deeper understanding of the driving mechanisms of lncRNAs in HCC development, progression, and resistance to chemotherapy. They revealed the importance of *DUXAP8* in HCC progression, which can help identify new prognostic indicators and therapeutic targets for HCC patients.

## Materials and Methods

### Cell Lines and Cell Culture

Standard human hepatocytes THLE-3 and HCC cell lines MHCC-97H, Huh7, HCC-LM3, Bel-7405, SNU-449, SK-Hep-1, and SNU-182 were purchased from the American Type Culture Collection or the Cell Bank of the National Collection of Authenticated Cell Cultures (Shanghai, China). The conventional culture of HCC cell lines was performed (Li et al., 2015a). Cells were cultured in appropriate culture media containing 10% fetal bovine serum (FBS; Gibco) supplemented with penicillin (100 U/ml) and streptomycin (100 μg/ml). All cell lines were cultured at 37°C in an incubator using 5% CO_2_/95% air and 100% humidity to minimize the chances of bacterial contamination. Cells were passaged every 1–2 days to maintain logarithmic growth.

### Patients and Specimens

All studies involving human samples were reviewed and approved by the Ethics Committee of the Second Hospital of Jilin University. The study protocol conformed to the ethical standards of the Declaration of Helsinki. Written informed consent was also obtained from all patients according to the Helsinki Declaration. Liver cancer and pericarcinomatous tissues were obtained from HCC patients at the Second Hospital of Jilin University (*n* = 79). The examined clinicopathological features included age, sex, number of tumor nodules, etiology, serum alpha fetoprotein (AFP) level, cancer staging, tumor size, and the presence of vascular invasion. Cancer staging was based on the 6th edition of the International Union Against Cancer tumor–node–metastasis (TNM) Classification.

### Chemical Reagents and Antibodies

Lipofectamine 2000 transfection reagent and total RNA extractant (TRIzol) were purchased from Invitrogen (Grand Island, NY, United States); *P-CREB* antibodies were purchased from Cell Signaling Technology (Danvers, MA, United States); *P38 and P-P38* antibodies were purchased from Abcam Corporation (Cambridge, MA, United States); and all other antibodies were purchased from Proteintech (Rosemont, IL, United States). Unless otherwise stated, all other chemical reagents were purchased from Sigma-Aldrich (St. Louis, MO, United States).

### Reverse Transcription Quantitative Polymerase Chain Reaction (RT–qPCR)

Total RNA was extracted using the RNA Simple Total RNA Kit (TIANGEN, DP419), and RNA in the cytoplasm and nucleus was isolated using the Nuclear/Cytoplasmic Isolation Kit (BioVision, San Francisco, CA, United States) according to the manufacturer’s instructions. Complementary DNA (cDNA) was synthesized using the RevertAid First Strand cDNA Synthesis Kit (Thermo Scientific, #K1622) and Poly(A) Polymerase Reaction Buffer (NEB, M0276s) according to the manufacturer’s instructions. According to the manufacturer’s instructions, real-time qRT–PCR analysis was performed using the Platinum SYBR Green qPCR SuperMix-UDG kit (Life Technologies, Gaithersburg, MD, United States). Fold changes in RNA expression were quantified using the 2^−ΔΔCt^ method. The primer sequences used in this study are listed in Supplementary Table S1.

### Immunohistochemistry (IHC)

The paraffin sections of tissues were dewaxed in xylene and hydrated using graded ethanol. The heat-mediated and antigen repair citrate (0.01 M, pH 6.0) was used in performing the IHC experiment. Endogenous peroxidase activity was blocked using 3% H_2_O_2_ at room temperature for 15 min. Thereafter, the sections were incubated with goat serum for 1 h to block the nonspecific binding site and then incubated overnight at 4°C using the primary antibody. The sections were then re-warmed at room temperature for 30 min, rinsed three times with phosphate-buffered saline (PBS) for 5 min each, and incubated with horseradish peroxidase (HRP)-labeled secondary antibody at room temperature for 1 h. Each section was rinsed thrice again with PBS for 5 min each. Color development was conducted using the HRP DAB kit (Thermo Science, Shanghai, China). Nuclei were re-stained with hematoxylin, and sections were sealed with neutral glue. Images were obtained using an Olympus X71 inverted microscope (Olympus Corp., Tokyo, Japan).

### Western Blot

Tissues or cells were homogenized and lysed using a lysis buffer. After the protein concentration was determined using the bicinchoninic acid method, β-mercaptoethanol, and bromophenol blue were added to the sample buffer for electrophoresis. Proteins were separated through 10% polyacrylamide gel electrophoresis and then transferred to polyvinylidene difluoride membranes (Bio-Rad, Shanghai, China). The membranes were incubated overnight at 4°C with primary antibodies. After incubation with secondary antibodies for 2 h at room temperature, the reaction bands were visualized using an enhanced chemiluminescence system, and the intensity of the bands was quantified using an image analysis system.

### Methylated RNA Immunoprecipitation qPCR (MeRIP–qPCR)

The level of m6A in *DUXAP8* was determined using MeRIP–qPCR. The intracellular RNA was first extracted using the TRIzol reagent and then bound to protein A/G magnetic beads with anti-m6A antibody or immunoglobulins (IgG; Cell Signaling Technology) (3 μg), and mixed with 100-μg total RNA in an immunoprecipitation buffer containing RNase/protease. The m6A-modified RNA was eluted twice with 6.7-mM N6-methyladenosine 5′-monophosphate disodium salt at 4°C for 1 h. RT–qPCR analysis was subsequently performed to determine the extent of m6A enrichment on *DUXAP8*.

### Luciferase Assay

The complementary DNA fragment containing wild-type or mutant *DUXAP8* fragment/*MAPK1* 3′ untranslated region (UTR) was subcloned to the downstream of luciferase in the pGL3-basic luciferase reporter gene (Promega, Beijing, China). The firefly luciferase gene containing wild-type or mutant *DUXAP8* fragment/*MAPK1* was cotransfected with an empty vector or miR-584-5p mimics, and luciferase activity was measured using the Dual-Luciferase Kit (Promega, Beijing, China) 48 h after transfection.

### RNA Immunoprecipitation (RIP) Analysis

According to the manufacturer’s instructions, the Magna RIPTM RNA Binding Protein Immunoprecipitation Kit (Millipore, United States) was used. Briefly, cell extracts were immunoprecipitated with antibodies against AGO2 or IgG and magnetic beads at 4°C for 6 h. The proteins in the complex were removed using 0.1% SDS/proteinase K (0.5-mg/ml, 55°C for 30 min), and immunoprecipitated proteins and RNA were detected using Western blot and RT–qPCR, respectively.

### RNA Pull-Down Experiments

The Pierce Magnetic RNA-Protein Pull-Down Kit (Thermo Fisher Scientific, 20,164) was used according to the manufacturer’s instructions. Briefly, cell lysates were treated with RNAase-free DNAase I and then incubated with the treated cell lysate, streptavidin-labeled magnetic beads, and a biotin-labeled *DUXAP8* probe. The magnetic beads could capture proteins/miRNAs that interact with *DUXAP8*. A Pierce™ RNA 3′ End Desthiobiotinylation Kit (Thermo, 20,163) was used for *DUXAP8* biotinylation labeling. The proteins and RNA in the captured protein-RNA complexes were analyzed using Western blot and RT–qPCR.

### Establishment of a Subcutaneous Xenotransplanted Tumor Model in Mice

Four-week-old BALB/c nude mice were purchased from the Experimental Animal Center of Jilin University (Changchun, China). All experimental animal protocols were performed according to the “Guide for the Care and Use of Laboratory Animals” issued by the National Institutes of Health. Experimental animal protocols for this study were reviewed and approved by the Animal Experiment Ethics Committee of the First Hospital of Jilin University. During modeling, 2 × 10^6^ Huh7 cells with silenced *DUXAP8* expression or negative control Huh7 cells were injected subcutaneously into the lateral abdominal region of each mouse. The tumor volume was measured weekly and calculated as V = (length × width^2^)/2. The tumors were then excised and weighed 4 weeks later.

### Statistical Analysis

All values were expressed as mean ± standard deviation. Comparisons between groups were made using a t-test or one-way analysis of variance. Qualitative data were analyzed using the chi-square test. Linear regression analysis was conducted for correlations between gene expression levels. Statistical analyses were performed using GraphPad Prism v8.0 (GraphPad, Inc., United States) and Statistical Software Package for Social Sciences (v 22.0; SPSS, Inc., Chicago, IL, United States). Differences were statistically significant when *p* < 0.05.

## Results

### High Expression of *DUXAP8* in an Animal Model of Patient-Derived Xenograft (PDX) HCC Treated With Sorafenib

**FIGURE 1:**
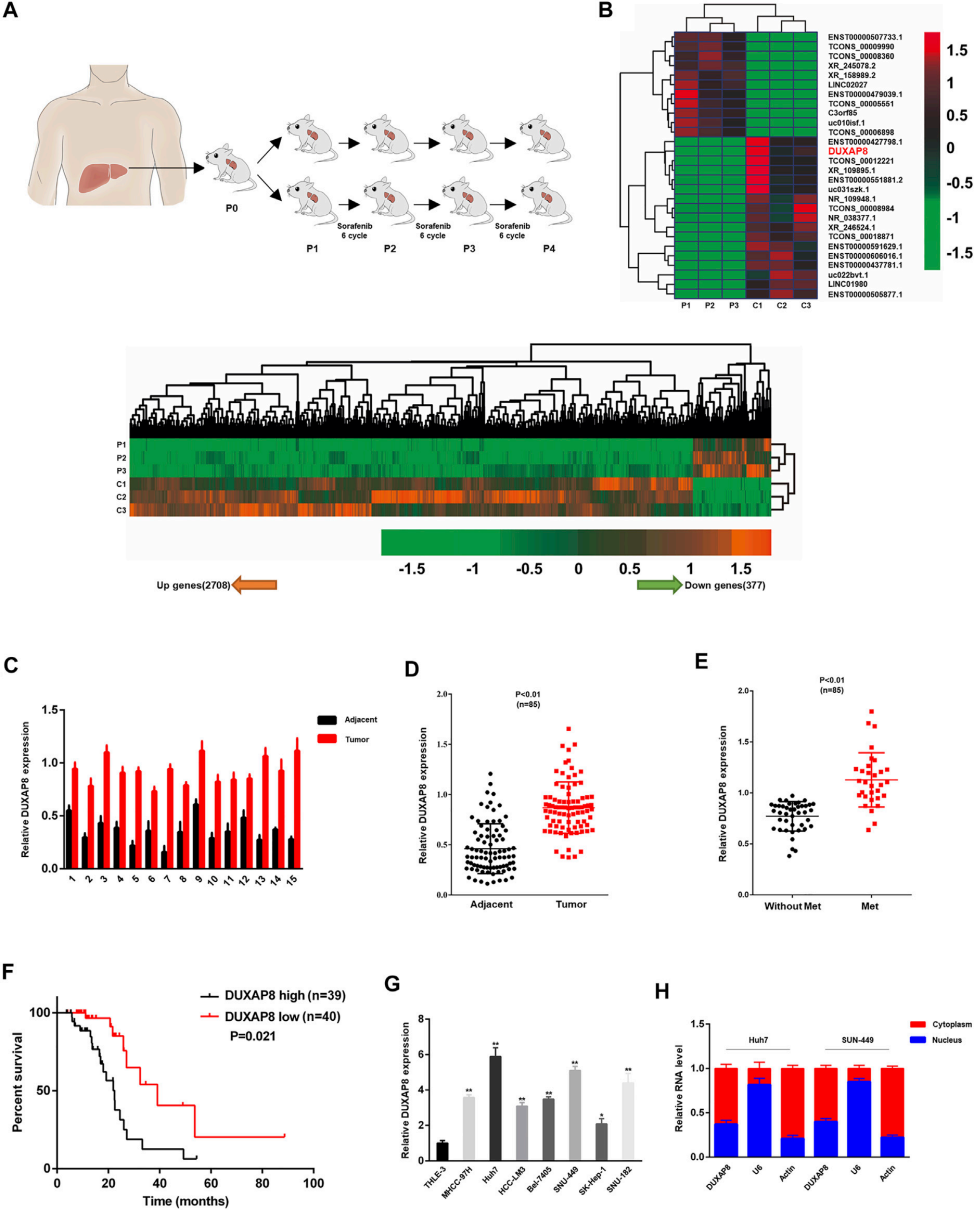
*DUXAP8* is highly expressed in sorafenib-treated patient-derived xenograft (PDX) hepatocellular carcinoma model, and its upregulation predicts poor prognosis of HCC. **(A)** Experimental procedure of the sorafenib-treated PDX HCC model. **(B)** The heat map indicates the differential expression of lncRNAs in sorafenib-treated and control P4-PDX mice. **(C)** Comparison of *DUXAP8* expression in liver cancer tissues of 15 HCC patients with paired pericarcinomatous normal tissues. **(D)** Comparison of *DUXAP8* expression in HCC tissues of 85 HCC patients with paired pericarcinomatous normal tissues. **(E)** Relationship between *DUXAP8* expression in liver cancer tissues of 85 patients and the presence of distant metastasis. **(F)** Kaplan-Meier analysis of the correlation between *DUXAP8* expression levels and HCC prognosis in 79 patients. **(G)** Comparison of *DUXAP8* expression in HCC cell lines and THLE-3 cells. **(H)** RT–qPCR assay of the distribution of *DUXAP8* in the nucleus and cytoplasm of HCC cells. Data are expressed as mean, *p < 0.05, **p < 0.01.

Sorafenib is an oral multi-kinase inhibitor. As the standard FDA-approved targeted therapy for HCC, sorafenib exhibits a survival benefit and has dramatically improved the prognosis of HCC patients, especially in patients with advanced HCC. However, acquired or intrinsic chemotherapy resistance severely affects its overall efficacy in treating HCC (Liu et al., 2019; Wei et al., 2019; Xiang et al., 2019). To clarify the molecular mechanism of drug resistance to sorafenib in HCC, a PDX model for sorafenib treatment was first established, in which surgically resected primary HCC tissue was finely trimmed and directly transplanted into mice with immunodeficiency. Tumor-bearing mice were treated with saline (vector) or sorafenib for several generations (Figure 1A). Differentially expressed lncRNAs between P4-PDX treated with vector or sorafenib were identified using lncRNA sequencing analysis (Figure 1B). Among identified lncRNAs, the significantly upregulated *DUXAP8* (ENST00000607933.1) was selected to investigate its role in HCC and chemosensitivity further (Supplementary Figure S1A).

It was confirmed by RT–qPCR that *DUXAP8* expression in HCC tissues was significantly higher than that of normal tissues adjacent to cancer, and the same results were obtained after expanding the sample size (Figures 1C,D). Correlation analysis with clinicopathological parameters showed that high levels of *DUXAP8* correlated with the TNM stage of cancer, tumor size, microvascular invasion, and distant metastasis (Figure 1E; Supplementary Table S2), and that HCC patients with high *DUXAP8* expression tended to have a poorer prognosis (Figure 1F). which is consistent with the results of the GEPIA database (Supplementary Figures S1B,C). We found that the expression of *DUXAP8* in seven HCC cell lines (MHCC-97H, Huh7, HCC-LM3, Bel-7405, SNU-449, SK-Hep-1, SNU-182) was significantly higher than its expression in the normal hepatocyte cell line THLE-3 (Figure 1G), and further confirmed that *DUXAP8* was mainly localized in the cytoplasm (Figure 1H). In conclusion, these findings suggested that *DUXAP8* is highly expressed in HCC tissues and positively correlates with the malignancy of HCC, and may function as a chemotherapy-resistant molecule in HCC.

### Overexpression of *DUXAP8* Promoted the Characteristics of Migration, Invasion, and Stemness of HCC Cells

**FIGURE 2:**
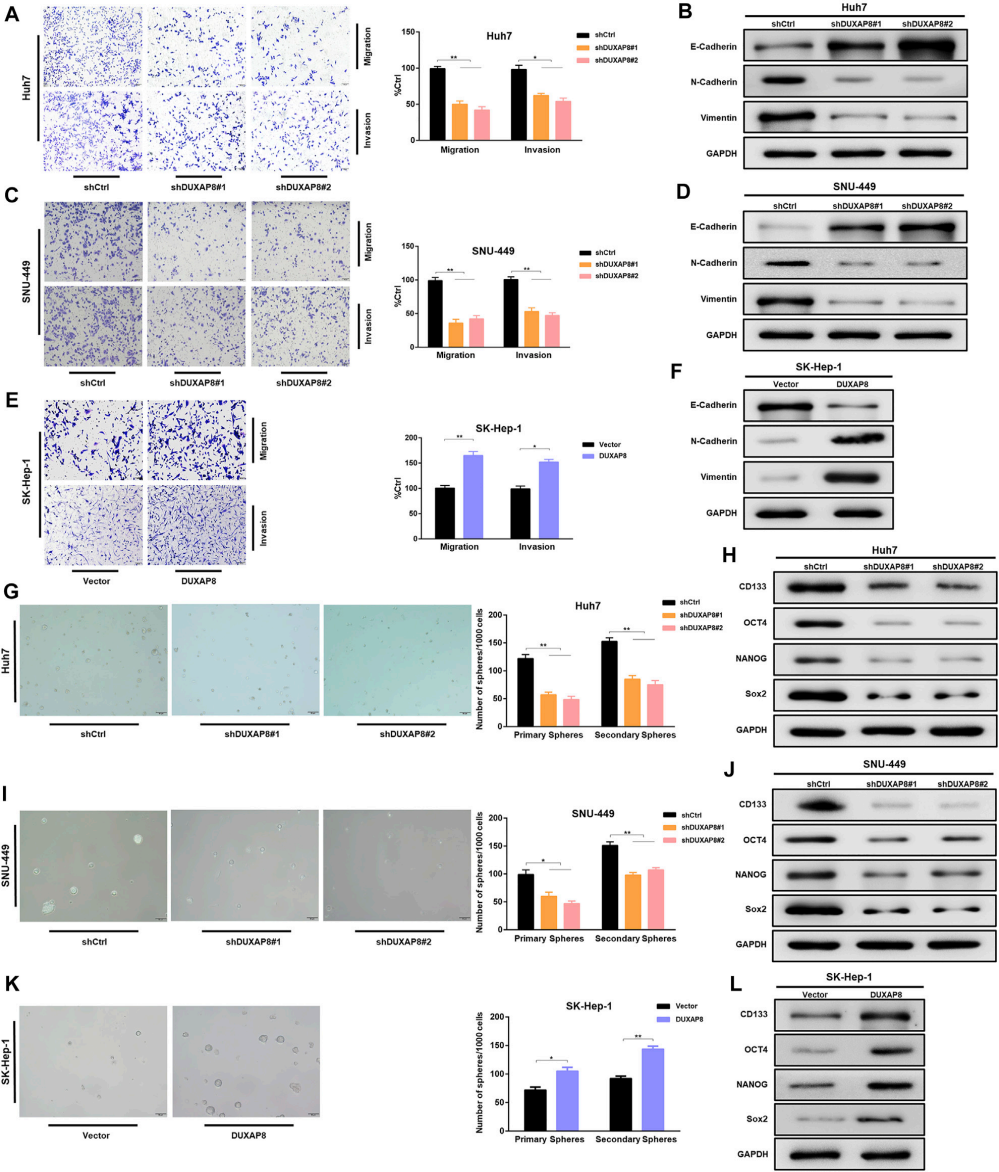
*DUXAP8* can regulate the characteristics of migration, invasion, and stemness of HCC cells. **(A,C,E)** DUXAP8 or shDUXAP8 and the control cells were subjected to Transwell migration and Matrigel invasion assays. **(B,D,F)** Western blot detects the expression of epithelial–mesenchymal transformation markers in *DUXAP8*-downregulated Huh7 and SNU-449 cells and *DUXAP8*-overxpressed SK-Hep-1 cells. **(G,I,K)** Statistical analysis of the primary and secondary spheroid-forming ability of *DUXAP8*-downregulated Huh7 and SNU-449 cells and *DUXAP8*-overexpressed SK-Hep-1 cells, with representative images showing secondary spheroid formation in these cells. **(H,J,L)** Western blot detects the expression levels of stemness-related genes in *DUXAP8*-downregulated Huh7 and SNU-449 cells and *DUXAP8*-overexpressed SK-Hep-1 cells. Data are expressed as mean, *p < 0.05, **p < 0.01.

To investigate the effect of *DUXAP8* on the biological behavior of HCC cells, shRNAs, and lentiviral vectors of *DUXAP8* mimics were transfected into *DUXAP8* high-expressing HCC cell lines (Huh7, SNU-449) and *DUXAP8* low-expressing HCC cell lines (SK-Hep-1, HCC-LM3), respectively, and the transfection efficiency was verified by RT-qPCR (Supplementary Figures S2A,B). To clarify whether *DUXAP8* affects the features of migration and invasion of HCC cells, Transwell assays were performed, which showed that the knockdown of *DUXAP8* significantly decreased the migratory and invasive ability of Huh7 and SNU-449 cells. In contrast, overexpression promoted the migration and invasion of SK-Hep-1 and HCC-LM3 cells (Figures 1A,C,E; Supplementary Figure S2C). Epithelial–mesenchymal transition (EMT) is closely related to the migration and invasion of tumor cells, and it was found through Western blot that when *DUXAP8* was knocked down, the epithelial marker E-cadherin increased and the mesenchymal marker N-cadherin and vimentin decreased in HCC cell lines (Figures 2B,D); when *DUXAP8* was overexpressed, it produced the opposite effect (Figure 2F; Supplementary Figure S2D), indicating that *DUXAP8* positively regulated EMT process in HCC cells. Altogether, *DUXAP8* overexpression promoted the migration and invasion of HCC cells.

Next, the effect of *DUXAP8* on the stem cell characteristics of HCC cells was investigated. The spheroid-forming ability was reduced when *DUXAP8* was knocked down in Huh7 and SNU-449 cells (Figures 2G,I). In contrast, *DUXAP8* overexpression significantly improved the spheroid-forming ability of SK-Hep-1 and HCC-LM3 cells (Figure 2K; Supplementary Figure S2E). The potential regulatory effects of *DUXAP8* on the expression of the cancer stem cell marker, *CD133*, and stem cell-related genes, including *OCT4*, *NANOG*, and *Sox2* were then investigated. It can be seen that *DUXAP8* knockdown significantly suppressed the expression of *CD133, OCT4, NANOG*, and *Sox2* (Figures 2H,J, Supplementary Figure S2G), while the expression of *CD133, OCT4, NANOG*, and *Sox2* was promoted when *DUXAP8* was overexpressed (Figure 2L, Supplementary Figures S2F,H). Thus, *DUXAP8* overexpression promoted the stem cell features of HCC cells.

### Overexpression of *DUXAP8* Promoted the Proliferation of HCC Cells and Reduced the Chemosensitivity of HCC Cells to Sorafenib

**FIGURE 3:**
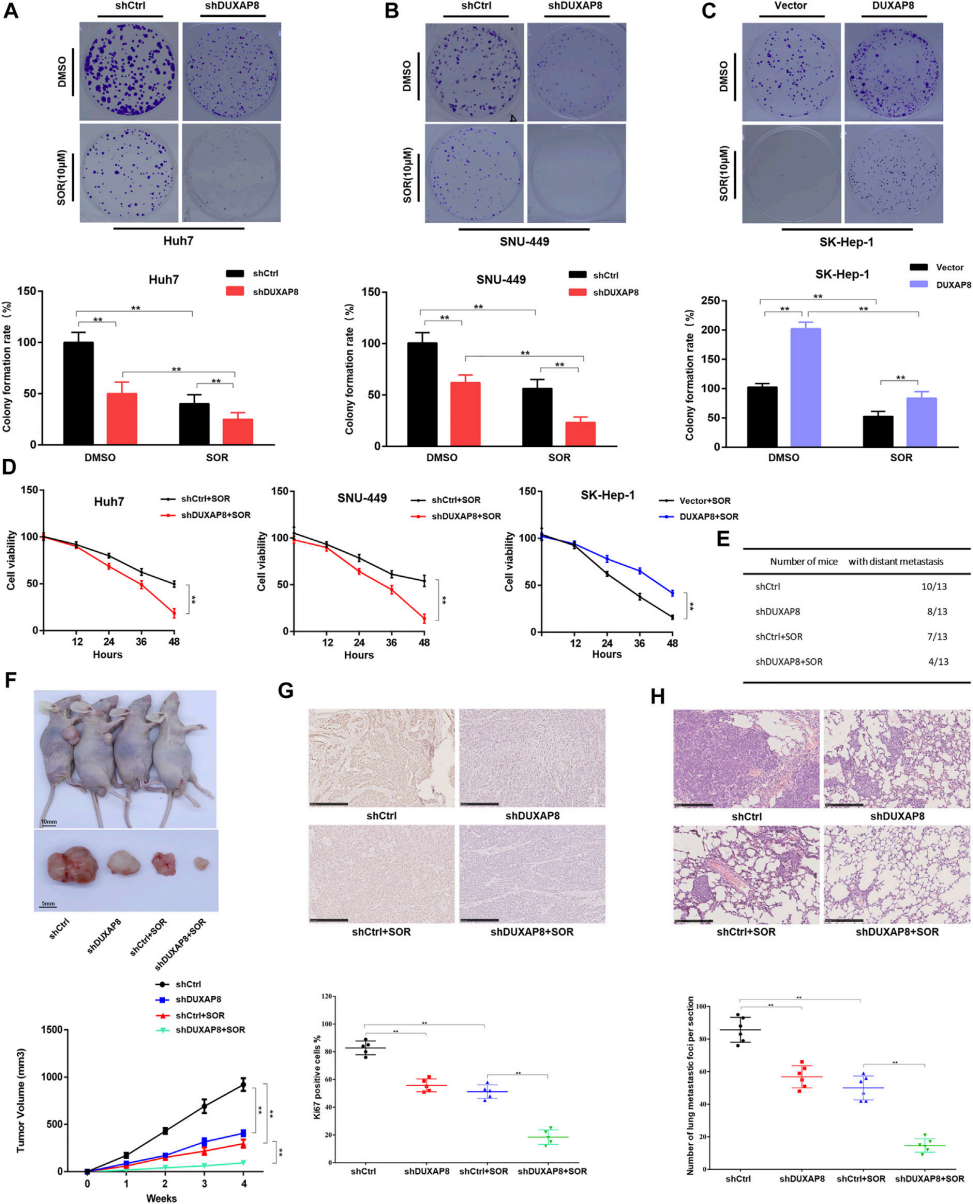
*DUXAP8* promotes the chemotherapy resistance of HCC to sorafenib *in vivo* and *in vitro*. **(A,B)** Colony formation of cells transfected with *shDUXAP8* after sorafenib treatment. **(C)** Colony formation of cells transfected with *DUXAP8*-overexpressing plasmid after sorafenib treatment. **(D)** Cell viability of Huh7/SNU-449 cells transfected with *shDUXAP8* and SK-Hep-1 cells transfected with *DUXAP8*-overexpressed plasmid under the effect of sorafenib wasanalyzedusingCCK8. **(E)** Summary table of pulmonary metastases. **(F)** Typical images of xenograft tumors formed by liver cancer cells transfected with shCtrl or *shDUXAP8* in sorafenib-treated nude mice. Tumor growth curves of HCC cells transfected with shCtrl or *shDUXAP8* in sorafenib-treated nude mice. **(G)** *Ki67* expression in xenograft tumors formed by shCtrl- or *shDUXAP8*-transfected HCC cells in sorafenib-treated nude mice **(H)** Number of metastatic foci per section in the lungs of nude mice treated with sorafenib after caudal vein injection of HCC cells transfected with shCtrl or *shDUXAP8*. Data are expressed as mean, **p < 0.01.

It was further investigated whether *DUXAP8* affected the chemosensitivity of HCC cells to sorafenib. It was observed that the knockdown of *DUXAP8* inhibited the proliferation ability of HCC cells and enhanced the chemosensitivity of HCC cells to sorafenib, while its overexpression promoted the proliferation of HCC cells and decreased the chemosensitivity of HCC cells to sorafenib (Figures 3A–D, Supplementary Figure S3A–C), indicating that *DUXAP8* mediated the development of chemotherapy resistance to sorafenib in HCC cells. Furthermore, consistent with the above *in vitro* findings, in *in vivo* experiments, knockdown of *DUXAP8* decreased the size and Ki67-positive rate of transplanted tumors in saline (NS) and sorafenib-treated nude mice (Figures 3F,G). Additionally, in the pulmonary metastasis model, it was found that knockdown of *DUXAP8* significantly inhibited the ability of pulmonary metastasis of HCC cells, as evidenced by the reduction in the number of mice with metastatic lung tumors and the decrease in the number of lung tumors in mice treated with NS or sorafenib (Figures 3E,H, Supplementary Figure S3D). In summary, these results suggested that overexpression of *DUXAP8* promoted the proliferation of HCC cells and reduced the chemosensitivity of HCC cells against sorafenib.

### Mettl3-Mediated m6A Modifications Involved in the Upregulation of *DUXAP8*

**FIGURE 4:**
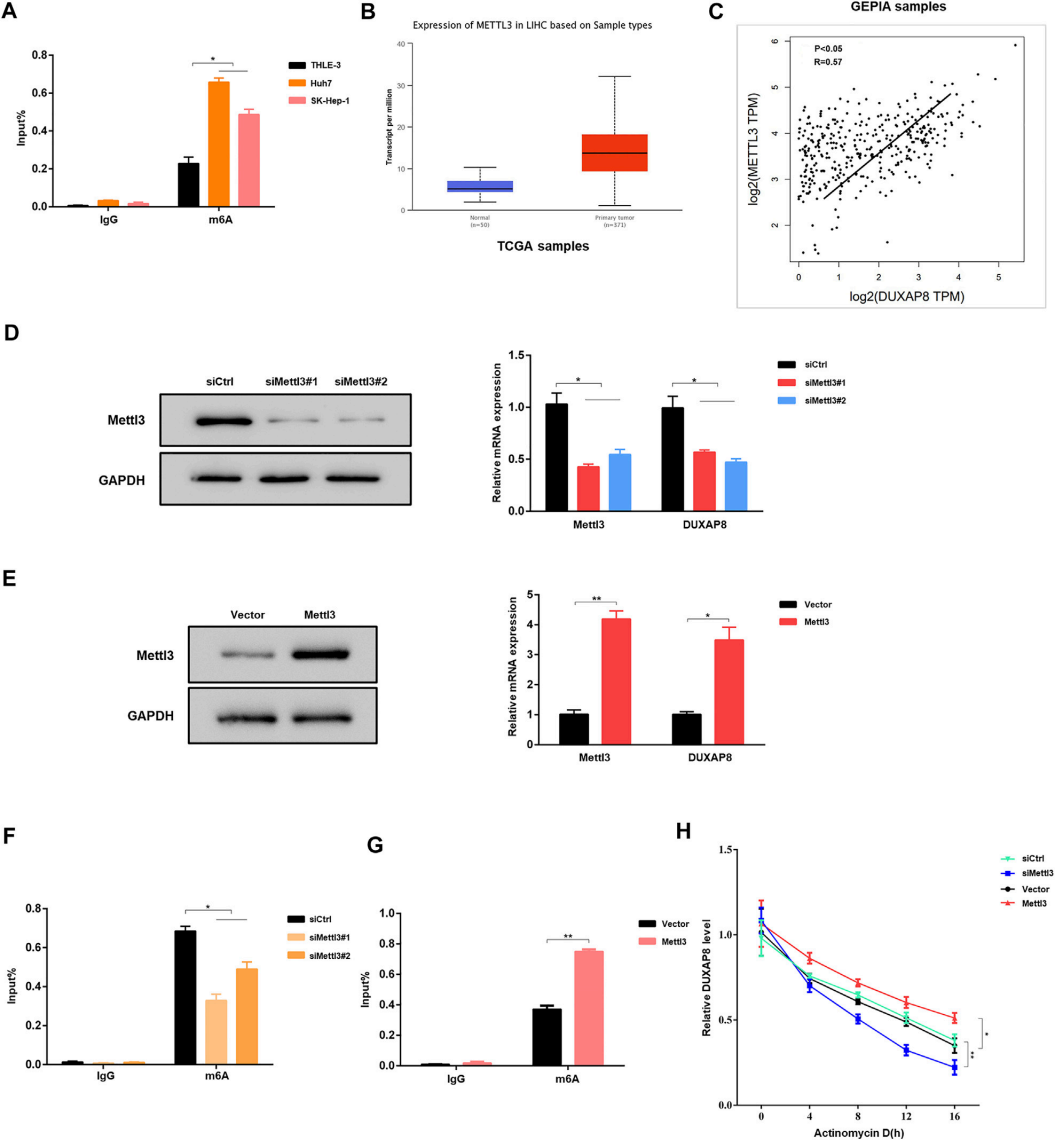
Mettl3-mediated m6A modifications are involved in the upregulation of *DUXAP8*. **(A)** MeRIP–qPCR results showed that the m6A enrichment of *DUXAP8* was higher in Huh7 and SK-Hep-1 cells than in THLE-3 cells. **(B)** The expression of methyltransferase 3 (Mettl3) in HCC was analyzed using the TCGA database. **(C)** The results of TCGA database showed that the expression level of *Mettl3* was positively correlated with *DUXAP8*. **(D,E)** Effect of Mettl3 knockdown and overexpression on *DUXAP8* expression. **(F,G)** Effect of *Mettl3* knockdown and overexpression on the degree of m6A enrichment of *DUXAP8*. **(H)** Effect of Mettl3 knockdown and overexpression on *DUXAP8* stability in the presence of dactinomycin. Data are expressed as mean, *p < 0.05, **p < 0.01.

Subsequently, the mechanism of *DUXAP8* upregulation in HCC cells was investigated. N6-methyladenosine (m6A) is the most common internal post-transcriptional modification in eukaryotic RNA (Wei et al., 1975; Schwartz et al., 2013), affecting RNA transcription, processing, translation, and metabolism. As a novel RNA epigenetic modification, the m6A modification plays an essential role in gene expression, including mRNA metabolism and other fundamental life processes and in the development of malignant tumors. RMBase (http://rna.sysu.edu.cn/rmbase/index.php) was used to find that *DUXAP8* has many m6A-binding sites. Therefore, it was hypothesized that the upregulation of *DUXAP8* in HCC might be associated with its m6A modification. The experimental results of MeRIP–qPCR verified the hypothesis that HCC cell lines (Huh7, SK-Hep-1) had a significantly higher level of m6A enrichment of *DUXAP8* than that of the normal hepatocyte line (THLE-3) (Figure 4A). Methyltransferase 3 (Mettl3) is a methyltransferase in m6A modification, which plays a vital role in mediating the m6A modification process in mammalian cells. By searching the Cancer Genome Atlas (TCGA) database, it was found that the expression level of Mettl3 was significantly higher in liver cancer tissues than in the corresponding pericarcinomatous tissues (Figure 4B), and there was a significant positive correlation between the expression levels of Mettl3 and *DUXAP8* (Figure 4C). When Mettl3 was silenced, it was observed that the expression level of *DUXAP8* also showed a significant decrease (Figure 4D); when Mettl3was overexpressed, the expression level of *DUXAP8* also indicated a corresponding increase (Figure 4E). It was also found that the m6A enrichment of *DUXAP8* in HCC cells decreased also when Mettl3 was silenced (Figure 4F), while the m6A enrichment of *DUXAP8* in HCC cells was correspondingly increased when Mettl3 was overexpressed (Figure 4G). The silencing of Mettl3 in the presence of dactinomycin, a drug that inhibits RNA synthesis, decreased the stability of *DUXAP8*, while Mettl3 overexpression produced the opposite result (Figure 4H). Altogether, as a methyltransferase in m6A modification, Mettl3 is essential to increase *DUXAP8* expression in HCC cells.

### The Effect of *DUXAP8* on the Malignant Phenotype and Chemosensitivity of HCC Through Competitive Binding to miR-584-5p

**FIGURE 5:**
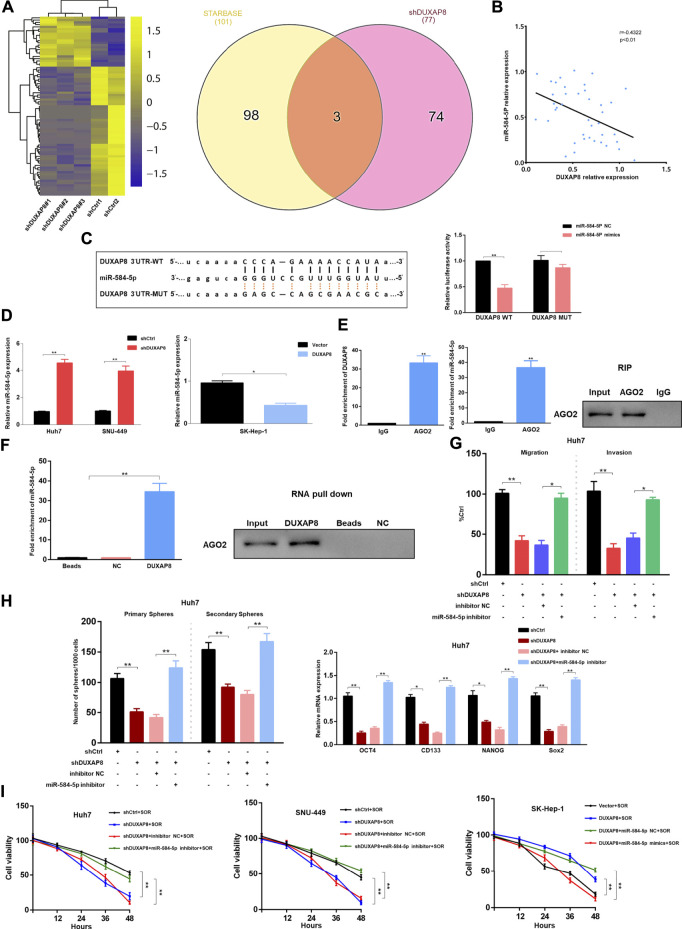
In HCC, *DUXAP8* acts as a molecular sponge for miR-584-5p, which downregulates miR-584-5p and affects the malignant phenotype and chemosensitivity of HCC. **(A)** The heat map demonstrating the differentially expressed microRNAs in HCC after *DUXAP8* downregulation, and Wayne plot showing the differentially expressed microRNAs after the intersection of RNA sequencing results with the bioinformatics database. **(B)** Correlation analysis between the expression of *DUXAP8* and miR-584-5p. **(C)** Dual-luciferase reporter assay detecting the relative activity of luciferase in transfected HCC cells. **(D)** Effect of *DUXAP8* downregulation and overexpression on miR-584-5p expression in HCC cells. **(E)** RIP assay detecting the relative enrichment of *DUXAP8* and miR-584-5p in anti-IgG or anti-AGO2 specific immunoprecipitates. **(F)** RNA pull-down assay was used to detect the interrelationship between *DUXAP8*, miR-584-5p, and AGO2. **(G,H)** Effects of *DUXAP8* and miR-584-5p downregulation on the migration, invasion, primary and secondary spheroid-forming ability, and stemness-related gene expression levels in Huh7 cells. **(I)** The viability of HCC cells with downregulated *DUXAP8* and miR-584-5p under the effect of sorafenib. Data are expressed as mean, *p < 0.05, **p < 0.01.

Cytoplasmic lncRNAs are often assumed to be competitive endogenous RNAs that can competitively bind to microRNAs, thereby producing an “isolation” effect on these microRNAs, reducing their regulatory effect on target genes (Tay et al., 2014). Considering the localization of *DUXAP8* in the nucleus and cytoplasm (Figure 1H), it was hypothesized that *DUXAP8* also exerts oncogenic effects in HCC through the ceRNA mechanism. To test this hypothesis, RNA sequencing analysis of *DUXAP8*-knockdown HCC cells was conducted to screen out significantly upregulated microRNAs, and the bioinformatics database, Encyclopedia of RNA Interactomes (previously known as starBase v2.0) was used, to predict the microRNAs that can target and bind to *DUXAP8*. In this study, two microRNAs were intersected, and three microRNAs (miR-584-5p, miR-409-3p, miR-374b-5p) were screened. miR-584-5p was selected, which exhibited the largest fold change, for subsequent experimental validation (Figure 5A, Supplementary Figure S4A). MiR-584-5p has been shown to be involved in regulating multiple malignant tumors (Xiang et al., 2015; Zhang et al., 2020), and it was found that in HCC tissues, *DUXAP8* showed a significant negative correlation with miR-584-5p expression (Figure 5B). Additionally, the overexpression of miR-584-5p could significantly reduce the luciferase activity of *DUXAP8* WT, but it had no effect on the luciferase activity of *DUXAP8* MUT (Figure 5C). The expression of miR-584-5p was significantly increased in HCC cells after the knockdown of *DUXAP8*, but miR-584-5p expression was significantly reduced when *DUXAP8* was overexpressed (Figure 5D). In the RIP, it was found that *DUXAP8* and miR-584-5p were highly enriched in AGO2 precipitation (Figure 5E). In the results of RNA pull-down assay, it was found that miR-584-5p and AGO2 were highly enriched in the biotin-labeled *DUXAP8* group (Figure 5F). The above experimental results suggested that *DUXAP8* acted as a molecular sponge for miR-584-5p in HCC through the ceRNA mechanism.

Next, it was investigated whether *DUXAP8* affects the malignant phenotype of HCC by competitively binding to miR-584-5p. When *DUXAP8* was knocked down, it significantly reduced the migration and invasion ability of Huh7 and SNU-449 cells while decreasing their spheroid-forming ability, and inhibited the expression of *CD133, OCT4, NANOG*, and *Sox2*, although such inhibitory effects of *DUXAP8* could be reversed using the knockdown of miR-584-5p (Figures 5G,H, Supplementary Figure S4B–D). When *DUXAP8* was overexpressed, it promoted the migration, invasion, and spheroid-forming ability of SK-Hep-1 cells and the expression of *CD133, OCT4, NANOG*, and *Sox2*. And this promotion effect could also be reversed through the overexpression of miR-584-5p (Supplementary Figure S4E–G).

As shown in Figure 5I, the knockdown of *DUXAP8* enhanced the chemosensitivity of HCC cells to sorafenib, while *DUXAP8* overexpression decreased the chemosensitivity of HCC cells to sorafenib, both results could be reversed by the knockdown and overexpression of miR-584-5p, respectively. In conclusion, *DUXAP8* could competitively bind to miR-584-5p through the ceRNA mechanism, thus affecting the malignant phenotype and chemosensitivity of HCC.

### miR-584-5p Affects the *MAPK/ERK* Pathway by Targeting *MAPK1*, a Protein Involved in *DUXAP8* Regulation of the Malignant Phenotype and Chemosensitivity in HCC

**FIGURE 6:**
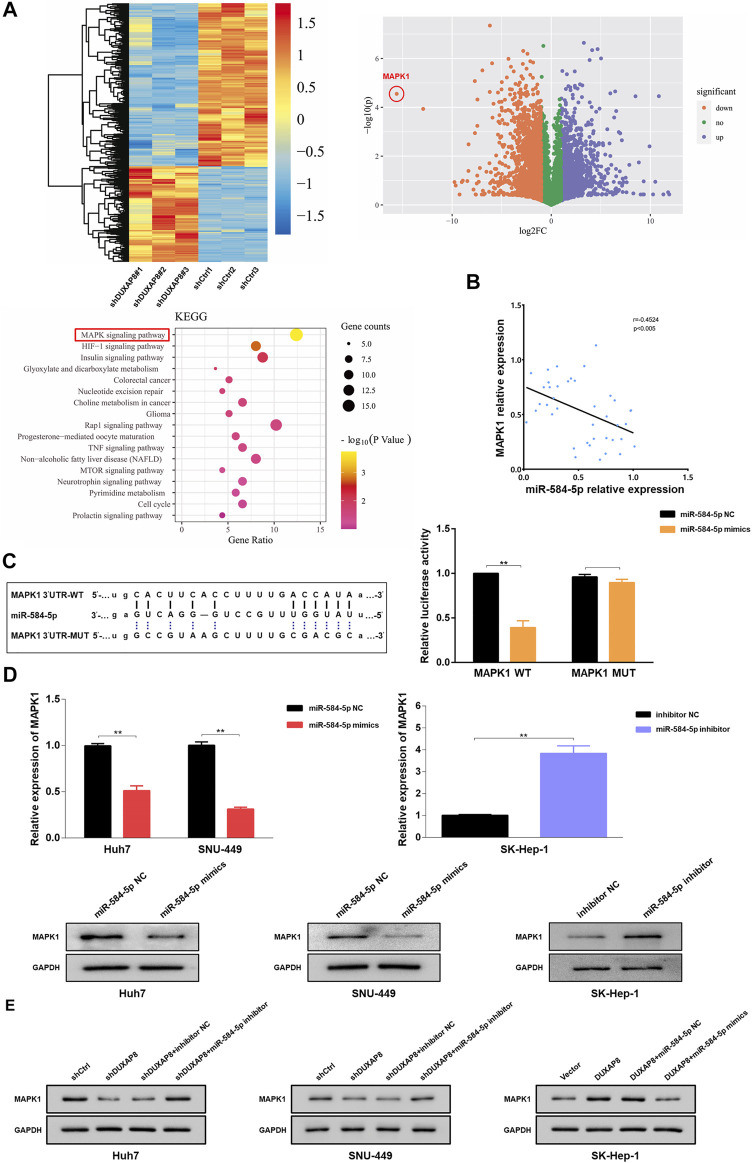
miR-584-5p targets *MAPK1* in HCC. **(A)** The heat map and volcano plot demonstrating the differentially expressed genes in HCC after *DUXAP8* downregulation, and Kyoto Encyclopedia of Genes and Genomes analysis of the main associated pathways. **(B)** The result of the correlation analysis showing that miR-584-5p was negatively correlated with MAPK1 expression. **(C)** Dual-luciferase reporter assay detecting the relative activity of luciferase in transfected HCC cells. **(D)** Effect of miR-584-5p downregulation and overexpression on *MAPK1* expression in HCC cells. **(E)** Effect of down-regulation or overexpression of *DUXAP8* and miR-584-5p on *MAPK1* expression in HCC during co-transfection. Data are expressed as mean, **p < 0.01.

To investigate the mechanism by which *DUXAP8* affects the malignant phenotype and chemotherapy resistance of HCC, RNA sequencing analysis of *DUXAP8*-knockdown HCC cells was performed. The results showed significant differences in the number of genes regulated by *DUXAP8* expression, and the *MAPK/ERK* pathway was the predominantly relevant signaling pathway (Figure 6A). *MAPK1* (mitogen-activated protein kinase 1, *ERK2*) is an essential biomarker of the *MAPK/ERK* pathway and can serve as a binding site for numerous biochemical signals (Jung et al., 2016; Wu et al., 2016). It has been shown that the activation of the *MAPK*/*ERK* pathway is closely associated with the invasion and migration, proliferation, drug resistance, and glycolysis in HCC (Liet al., 2020a; Li et al., 2020b; He et al., 2020). Therefore, it was hypothesized that miR-584-5p regulates the malignant phenotype and chemosensitivity of HCC by targeting *MAPK1* and affecting the *MAPK/ERK* pathway. it was that *MAPK1* negatively correlated with miR-584-5p expression in HCC tissues (Figure 6B), and the overexpression of miR-584-5p could significantly reduce the luciferase activity of *MAPK1* WT, but had no effect on the luciferase activity of *MAPK1* MUT (Figure 6C). Overexpression of miR-584-5p significantly reduced the expression of *MAPK1* of Huh7 and SNU- 449, whereas its knockdown produced the opposite result in SK-Hep-1 (Figure 6D). We also found that the knockdown of *DUXAP8* significantly reduced *MAPK1* expression in Huh7 and SNU-449 cells, however, this effect could be reversed by the downregulation of miR-584-5p. In contrast, overexpression of *DUXAP8* increased the expression of *MAPK1* in SK-Hep-1 cells, while overexpression of miR-584-5p reversed this effect (Figure 6E). The above experimental results showed that miR-584-5p could directly target *MAPK1* in HCC cells.

When miR-584-5p was overexpressed, it significantly reduced the migration and invasion ability of Huh7 and SNU-449 cells while reducing their spheroid-forming ability and also inhibited the expression of *CD133*, *OCT4*, *NANOG*, and *Sox2*, although this inhibitory effect could be reversed by *MAPK1* overexpression (Supplementary Figure S5). When miR-584-5p was knocked down, it promoted the migration, invasion, and spheroid-forming ability of SK-Hep-1 cells and the expression of *CD133*, *OCT4*, *NANOG*, and *Sox2*, and this effect could also be reversed by the knockdown of *MAPK1*. The overexpression of miR-584-5p enhanced the chemosensitivity of HCC cells to sorafenib, while its knockdown decreased the chemosensitivity of HCC cells to sorafenib, and both results could also be reversed through the overexpression and knockdown of *MAPK1*, respectively (Supplementary Figure S5).

**FIGURE 7:**
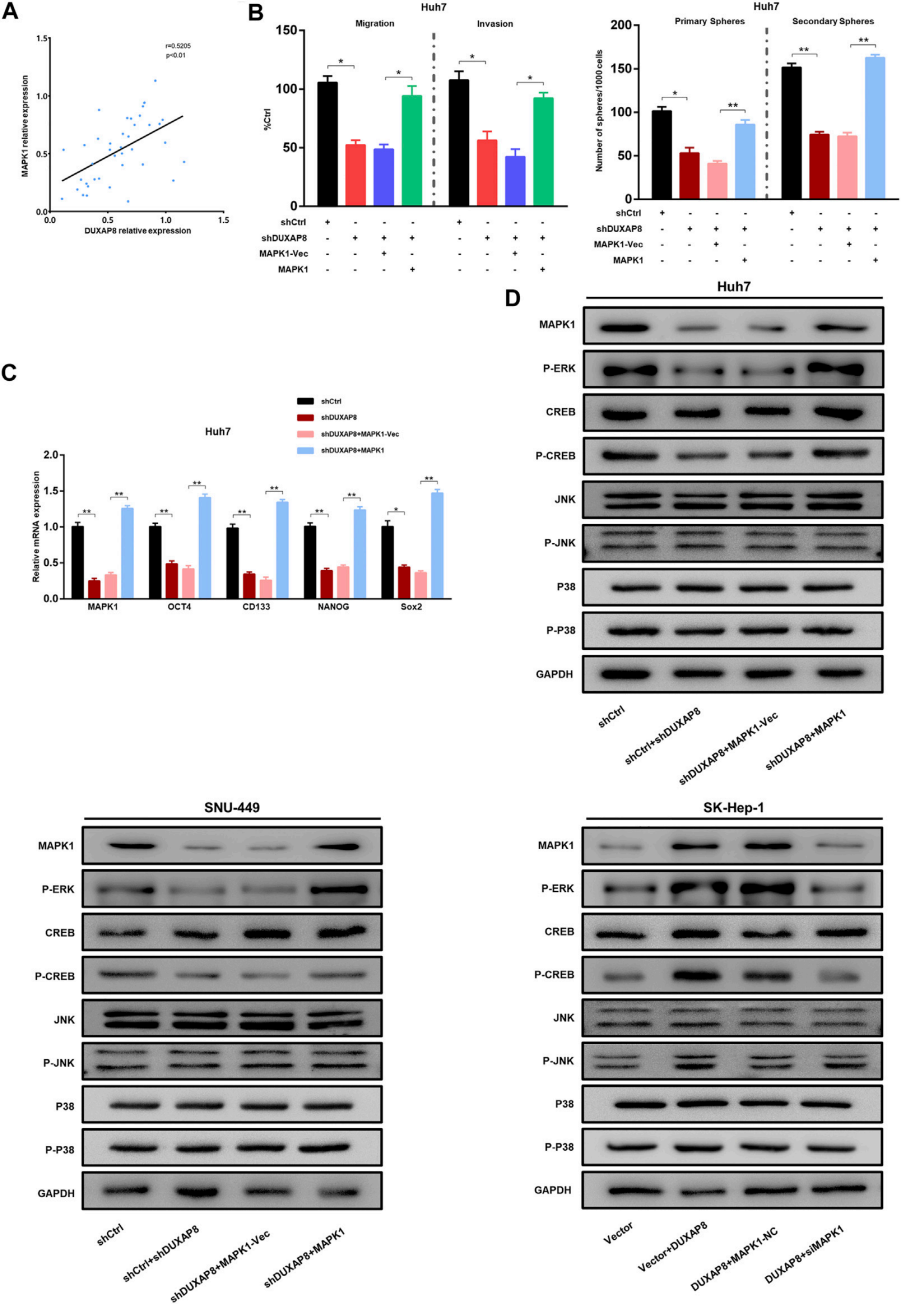
The *DUXAP8*/miR-584-5p/*MAPK1* axis regulates the malignant phenotype of HCC. **(A)** The result of the correlation analysis showing that *DUXAP8* was positively correlated with the expression of *MAPK1*. **(B)** Effects of *DUXAP8* downregulation and *MAPK1* overexpression on the migration, invasion, and primary and secondary spheroid-forming ability of Huh7 cells. **(C)** Effects of *DUXAP8* downregulation and *MAPK1* overexpression on the expression levels of stemness-related genes in Huh7 cells. **(D)** Effects of *DUXAP8* downregulation and *MAPK1* overexpression on *ERK/CREB/JNK/P38* and their phosphorylation levels in HCC cell lines. Data are expressed as mean, *p < 0.05, **p < 0.01.

Correlation analyses show that the expression of *DUXAP8* was positively correlated with *MAPK1* in HCC tissues (Figure 7A). Western blot results showed that the knockdown of *DUXAP8* downregulated the phosphorylation level of *ERK/CREB*, while the overexpression of *MAPK1* abolished the inhibitory effect of *DUXAP8* knockdown on the phosphorylation level of above proteins. However, there was no effect on the phosphorylation level of *JNK/p-38* (Figure 7D). This suggested that *DUXAP8* exerts its biological function by activating the *MAPK/ERK* pathway. To verify this conclusion, a rescue experiment was performed. When *DUXAP8* was knocked down, it significantly reduced the migration, invasion, and spheroid-forming ability of Huh7 and SNU-449 cells and the expression of *CD133, OCT4, NANOG*, and *Sox2*, but this inhibitory effect could be reversed by the overexpression of *MAPK1* (Figures 7B,C, Supplementary Figure S6). When *DUXAP8* was overexpressed, it promoted the migration, invasion and spheroid-forming ability of SK-Hep-1 cells and the expression of *CD133, OCT4, NANOG*, and *Sox2*, and this effect could also be reversed by the knockdown of *MAPK1* (Supplementary Figure S6). The knockdown of *DUXAP8* enhanced the chemosensitivity of HCC cells to sorafenib, while its overexpression reduced the chemosensitivity of HCC cells to sorafenib, and both results could be reversed by the overexpression and knockdown of *MAPK1*, respectively (Supplementary Figure S6).

**FIGURE 8:**
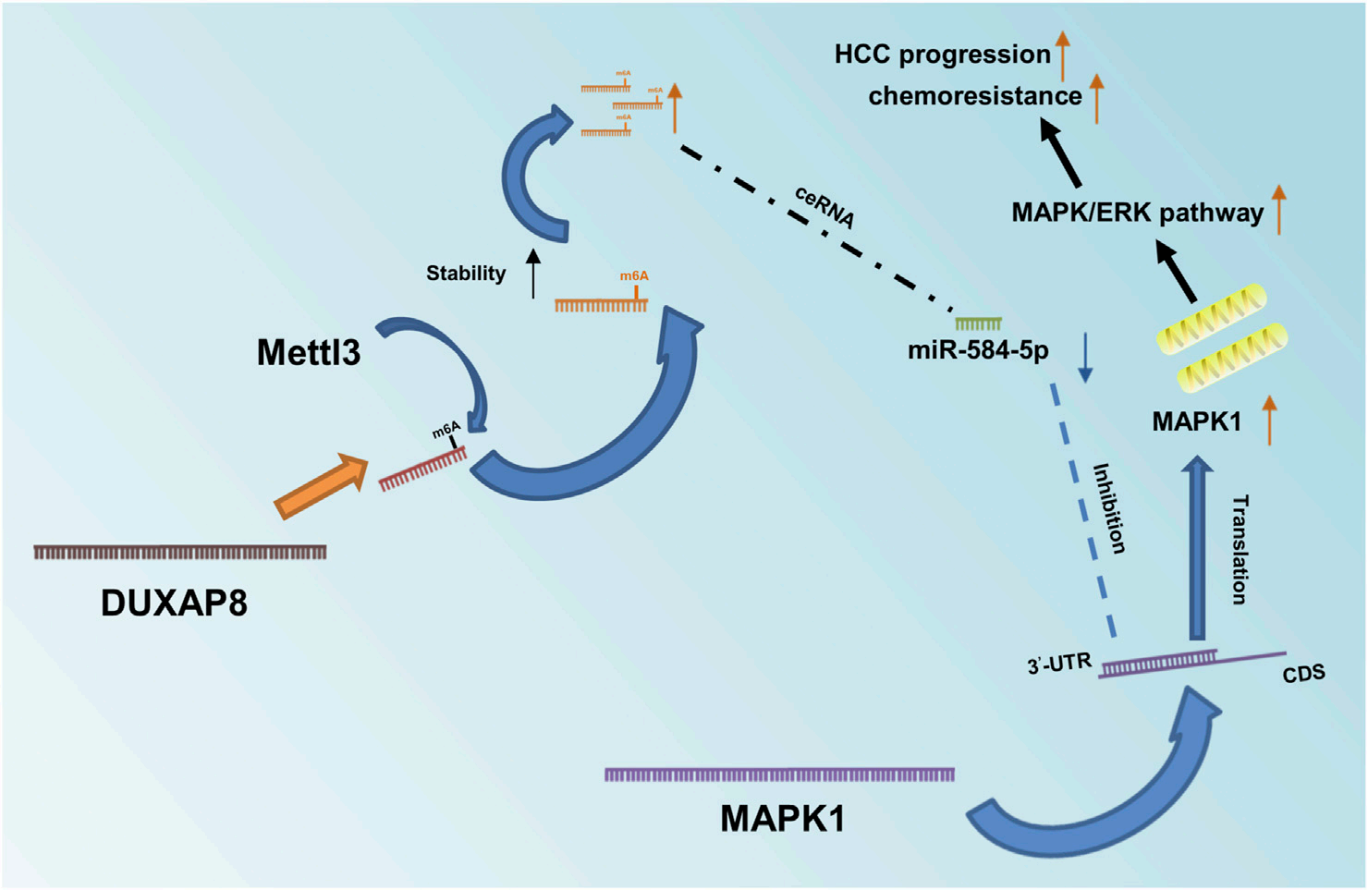
Diagram of *DUXAP8’*s role in HCC and its mechanism. Mettl3-mediated m6A methylation modification stabilizes and upregulates *DUXAP8*, which competitively binds to miR-584-5p through a ceRNA mechanism, thereby targeting *MAPK1* and activating the *MAPK/ERK* pathway to regulate the malignant phenotype and chemotherapy resistance of HCC.

In summary, *DUXAP8* competitively bound to miR-584-5P through the ceRNA mechanism (Figure 8), thus targeting *MAPK1* and activating the *MAPK/ERK* pathway to regulate the malignant phenotype and chemotherapy resistance of HCC.

## Discussion

The mechanisms of drug resistance in HCC are quite complex, involving the increased expression of drug efflux transporters (a type of protein that recognizes and pumps anticancer drugs out of tumor cells), intracellular redistribution of drugs, inactivation of the apoptosis signaling pathway, and activation of tumor stem cells (Butler et al., 2013; Salehan and Morse, 2013; Llovet et al., 2015). However, to date, the exact mechanism of drug resistance in HCC remains to be investigated.

In this study, the role of *DUXAP8* in promoting malignant phenotype and chemotherapy resistance in HCC was revealed, and it was confirmed for the first time that Mettl3-mediated m6A modifications were involved in the upregulation of *DUXAP8*. In summary, it was found that *DUXAP8* exhibited high expression in sorafenib-treated PDX models and was positively correlated with the TNM cancer stage, tumor size, microvascular invasion, and distant metastasis. The results of *in vivo* and *in vitro* experiments showed that *DUXAP8* is positively correlated with the proliferation, migration, invasion, and sorafenib resistance of HCC. *DUXAP8* upregulates *MAPK1* through competitively binding to miR-584-5p, which activates the *MAPK/ERK* pathway to perform its biological functions.

There is increasing evidence supporting that lncRNAs play an essential role in the occurrence and development of malignant tumors (Yuan et al., 2014; Li et al., 2015b; Chen et al., 2016; Yuan et al., 2016; Zhu et al., 2016). Until now, *DUXAP8* is highly expressed in various malignant tumors. *DUXAP8* can significantly inhibit the expression of *PLEKHO1* in gastric cancer, which enhances the proliferation and migration of tumor cells (Ma et al., 2017a). In glioma, the downregulation of *DUXAP8* inhibits the proliferation of tumor cells (Zhao et al., 2019). In non-small cell lung cancer (NSCLC), *DUXAP8* promotes tumor cell proliferation and invasion through epigenetically silencing *Egr1* and *RHOB* (Sun et al., 2017). In pancreatic carcinoma, *DUXAP8* promotes tumor growth through epigenetically silencing *CDKN1A* and *KLF2* (Lian et al., 2018). This study found significantly elevated expression of *DUXAP8* in HCC compared to that of the adjacent normal liver tissue, and this result is consistent with that in previous reports.

As a novel epigenetic RNA modification, m6A is closely related to the phenotype and mechanism of malignant tumors and plays an essential role in the self-renewal of tumor stem cells and the metabolism, recurrence and metastasis of various malignant tumors. The dynamic regulatory proteins modified by m6A include methyltransferases (Writers), demethylases (Erasers), and reading genes (Readers) (Zhao et al., 2017). The methyltransferases are multicomponent compounds consisting of *METTL3, METTL14, WTAP, KIAA1429, RBM15*, and *ZC3H13* (Knuckles et al., 2018). Recent studies (Jo et al., 2013; Bansal et al., 2014; Ma et al., 2017b; Cui et al., 2017; Visvanathan et al., 2018) have shown that m6A-related proteins are involved in the development and progression of different types of malignant tumors, such as acute myeloid leukemia, cholangiocarcinoma, glioblastoma, and hepatocellular carcinoma (HCC). In this study, it was demonstrated for the first time that *DUXAP8* is overexpressed in HCC because Mettl3-mediated m6A modification confers its stability.

Existing studies have demonstrated that lncRNAs are involved in the regulation of many diseases and play an essential role in the occurrence and development of malignant tumors. Some lncRNAs act as protein co-regulators by directly binding to helper proteins and regulating the expression of downstream tumor-associated genes (Chen et al., 2016; Yuan et al., 2016; Zhu et al., 2016). Another part of lncRNAs functions as competitive endogenous RNAs (ceRNAs) (Yuan et al., 2014; Li et al., 2015b). Such lncRNAs isolate microRNAs and regulate the expression of microRNA-targeted oncogenes or tumor suppressor genes through molecular sponge effects. Argonaute 2 (AGO2) is a core component of the microRNA (miRNA)-induced silencing complex (RISC), linking miRNAs and their mRNA target sites(Meister et al., 2004). And the mechanisms through which lncRNAs regulate the biological functions of malignant tumors largely depend on their subcellular localization (Statello et al., 2021). Considering that *DUXAP8* is mainly localized in the cytoplasm, while RNA sequencing analysis and bioinformatics analysis also indicate that *DUXAP8* has a putative binding site for miR-584-5p, it was therefore suspected that *DUXAP8* also affects HCC progression through a ceRNA mechanism. The luciferase reporter assay, RIP and RNA pull-down experiments confirmed this speculation. Meanwhile, the expression of miR-584-5p was correspondingly reversed when the expression of *DUXAP8* was changed. These data suggested that *DUXAP8* acts as a molecular sponge, which binds to miR-584-5p to perform its biological function. miR-584-5p was further investigated and it was found that miR-584-5p significantly inhibited the features of migration, invasion, and stemness of HCC and correspondingly enhanced its chemosensitivity to sorafenib. RNA sequencing analysis revealed that the downregulation of *DUXAP8* had a significant effect on the *MAPK/ERK* pathway, accompanied by the downregulation of *MAPK1*. Previous studies have reported that *MAPK/ERK* pathway is closely related to the invasion and migration, proliferation, drug resistance, and glycolysis in HCC (Li et al., 2020a; Li et al., 2020b; He et al., 2020). Additionally, *MAPK1* is an essential biomarker of the *MAPK/ERK* pathway and can serve as a binding site for numerous biochemical signals (Jung et al., 2016; Wu et al., 2016). Therefore, it was suggested that miR-584-5p might play an oncogenic role in HCC by targeting the downregulation of *MAPK1*, and subsequent findings supported this hypothesis. Through luciferase reporter assay, expression correlation analysis and cell function up-and downregulation assays in HCC, it was demonstrated that miR-584-5p could indeed directly affect the expression level of *MAPK1* in HCC cells. Meanwhile, the knockdown and overexpression of *MAPK1* could effectively reverse the effects of miR-584-5p knockdown and overexpression on the malignant phenotype and chemosensitivity of HCC cells to sorafenib. Then, it was further shown that the knockdown of *DUXAP8* inhibited the phosphorylation level of *ERK/CREB*, while the overexpression of *MAPK1* reduced the inhibitory effect of *DUXAP8* knockdown on the phosphorylation level of *ERK/CREB*. It was also suggested that *DUXAP8* exerts its biological function by activating the *MAPK/ERK* pathway.

In conclusion, this study revealed the role and potential mechanism of *DUXAP8* in promoting the malignant phenotype of HCC and chemoresistance. Mechanistically, *DUXAP8* endogenously competes for the binding of miR-584-5p through the ceRNA mechanism, reducing its inhibitory effect -on *MAPK1* and thus activating the *MAPK/ERK* pathway to promote the proliferation, invasive migration, stemness maintenance, and chemoresistance in HCC. These results have deepened the understanding of the driving mechanisms of lncRNAs in the development, progression, and chemotherapy resistance of HCC and provided greater insight into the importance of *DUXAP8* in HCC progression, which may help provide ideas for finding new prognostic indicators and therapeutic targets for HCC patients.

## Data Availability

The datasets presented in this study can be found in online repositories. The names of the repository/repositories and accession number(s) can be found below: https://doi.org/10.6084/m9.figshare.17049722.v1, https://doi.org/10.6084/m9.figshare.17049716.v1, https://doi.org/10.6084/m9.figshare.17049686.v1.
