# Supplementary figures and images for: m6A Modification-Mediated DUXAP8 Regulation of Malignant Phenotype and Chemotherapy Resistance of Hepatocellular Carcinoma Through miR-584-5p/MAPK1/ERK Pathway Axis
Source: Front Cell Dev Biol. 2021 Dec 9;9:783385. doi: 10.3389/fcell.2021.783385 (PMC8696125; doi:10.3389/fcell.2021.783385)

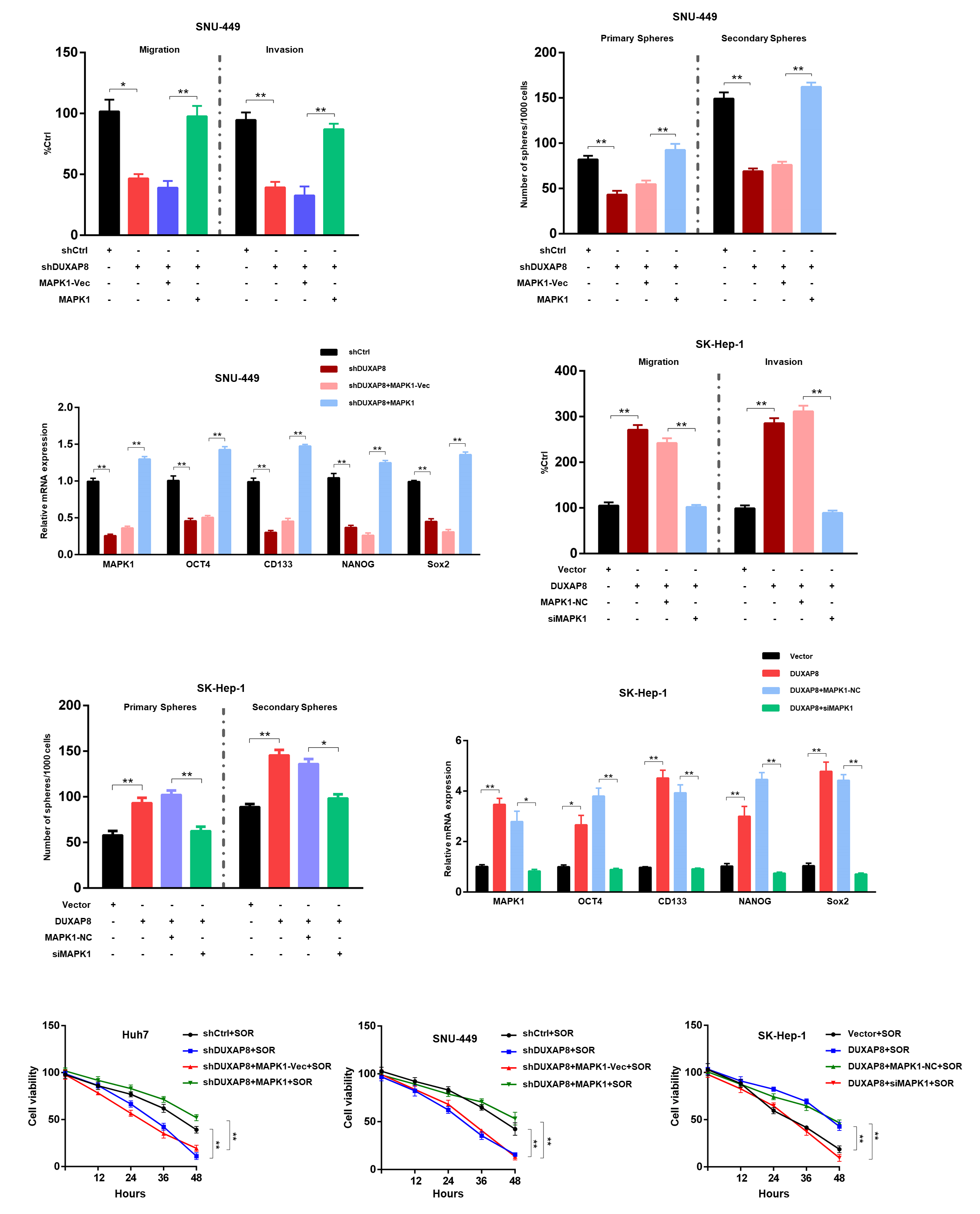

Supplement: Supplementary file 1 [file Image6.TIF]

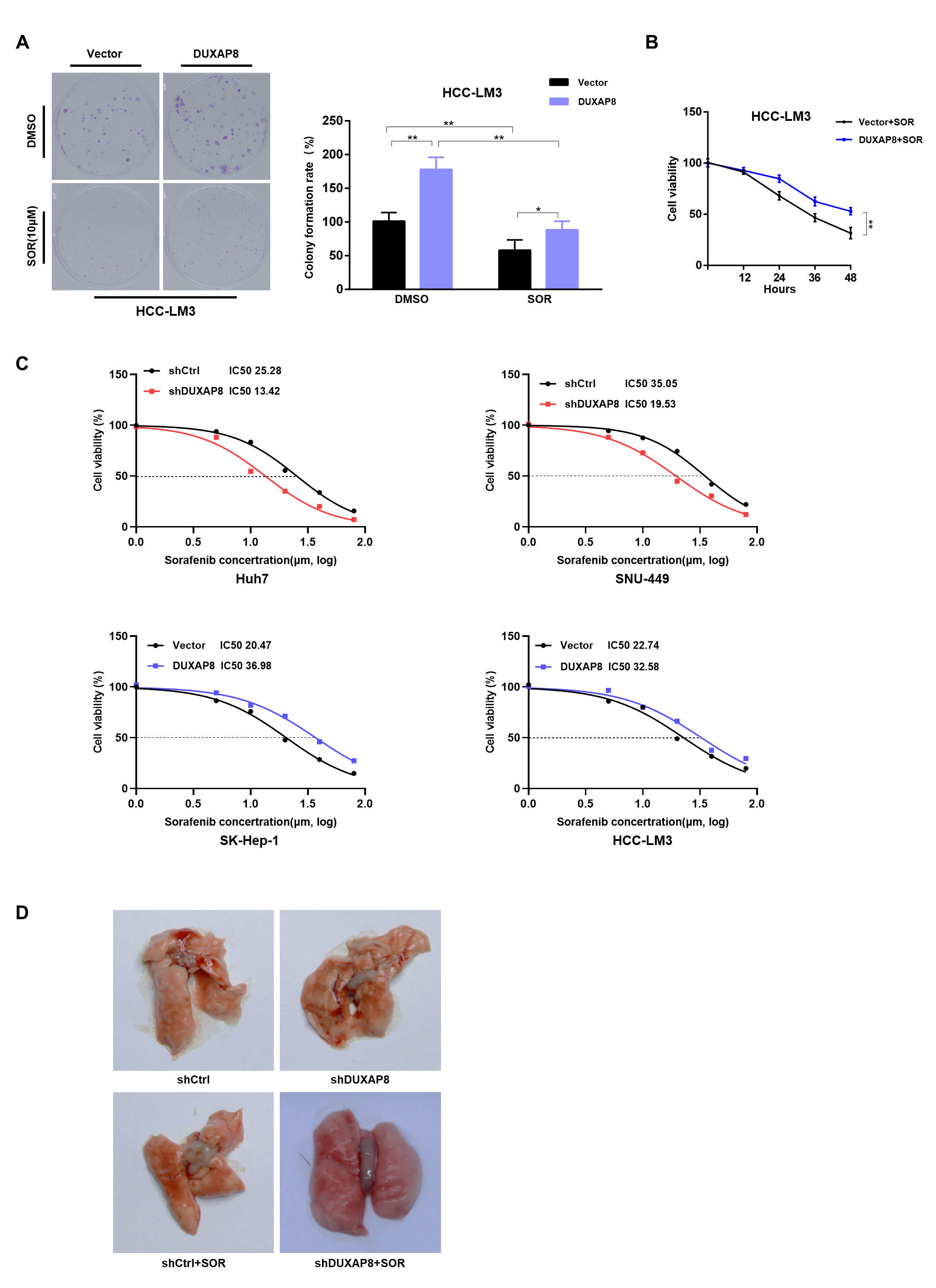

Supplement: Supplementary file 3 [file Image3.TIF]

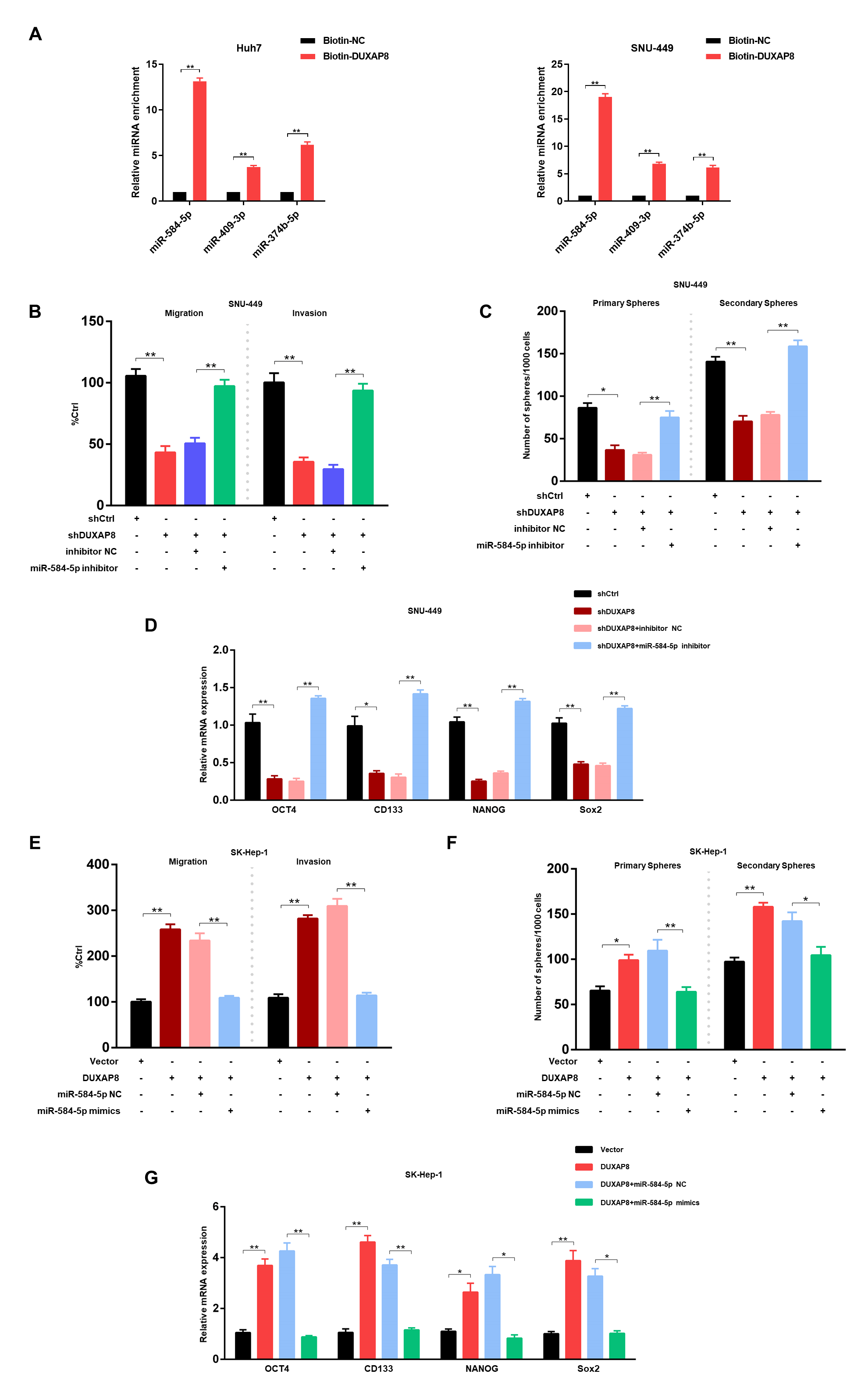

Supplement: Supplementary file 4 [file Image4.TIF]

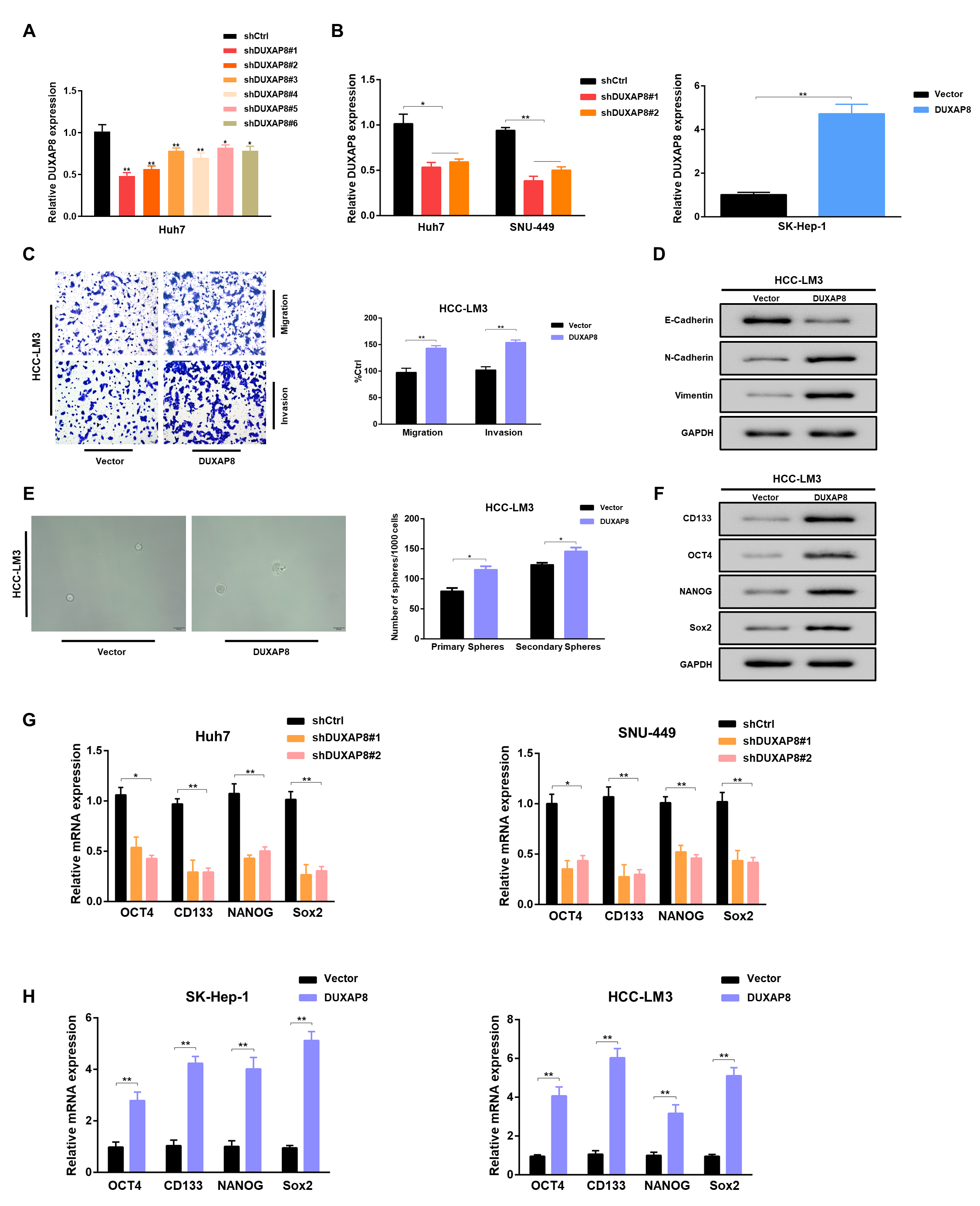

Supplement: Supplementary file 5 [file Image2.TIF]

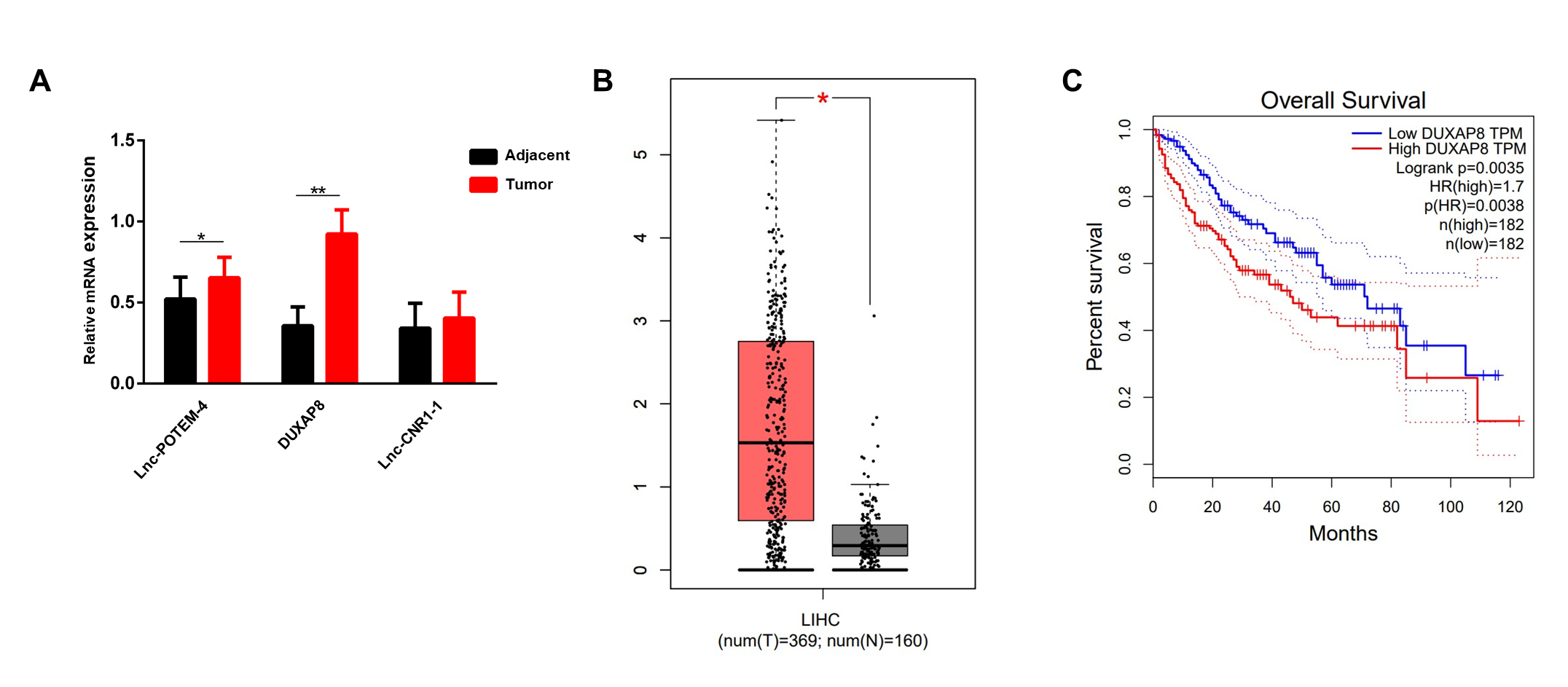

Supplement: Supplementary file 6 [file Image1.TIF]

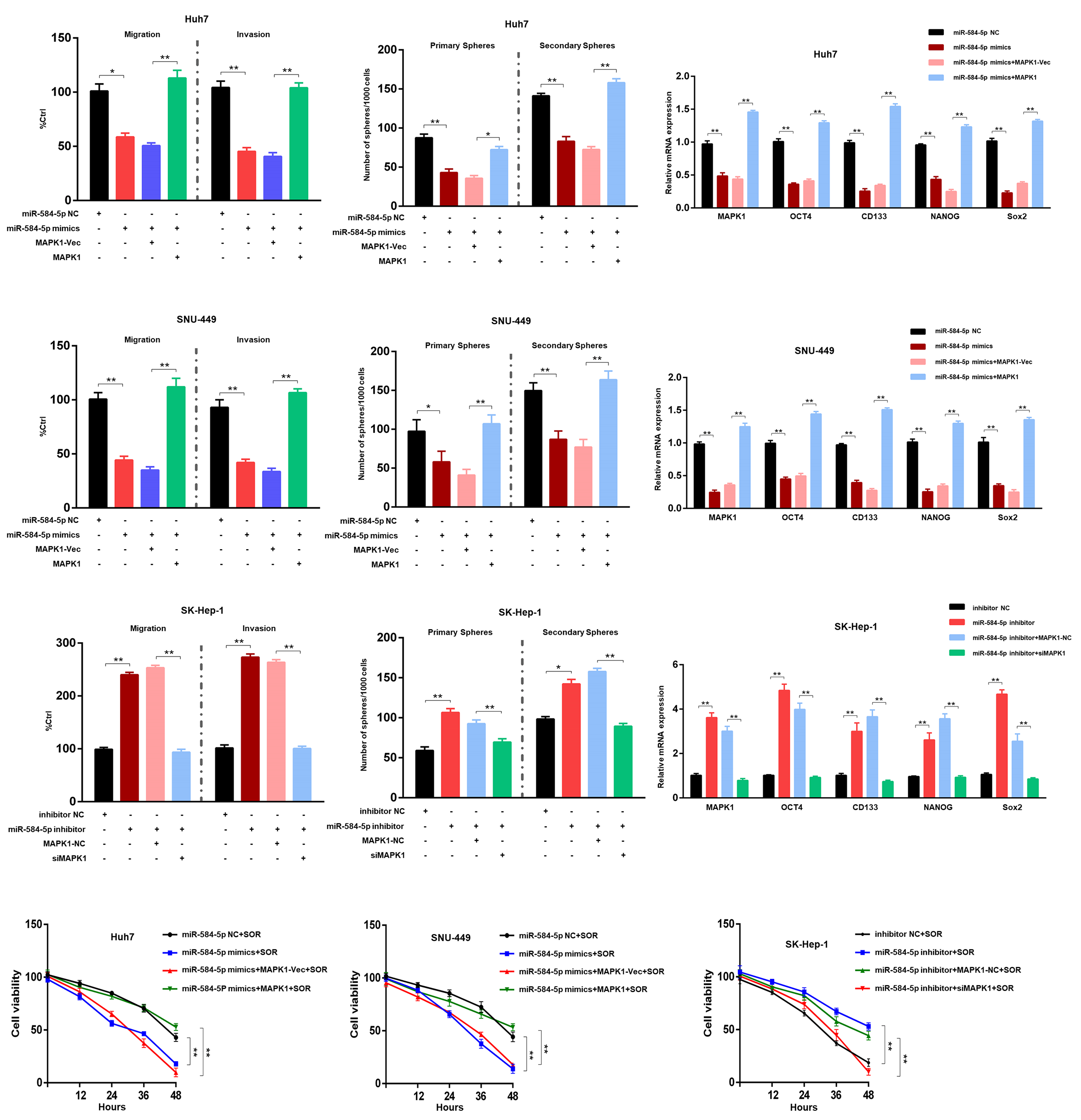

Supplement: Supplementary file 8 [file Image5.TIF]
