# Supplementary material for: m6A Modification-Mediated DUXAP8 Regulation of Malignant Phenotype and Chemotherapy Resistance of Hepatocellular Carcinoma Through miR-584-5p/MAPK1/ERK Pathway Axis
Source: Front Cell Dev Biol. 2021 Dec 9;9:783385. doi: 10.3389/fcell.2021.783385 (PMC8696125; doi:10.3389/fcell.2021.783385)
Supplement: Supplementary file 2 [file Table1.docx]

**Table S1 The sequences of primers for RT-qPCR**

| cDNA | Primers | Sequences |
| --- | --- | --- |
| DUXAP8 | forward | 5'-TCAGAGCGAGCTTCGGAGAA-3' |
|  | reverse | 5'-GCAGGGTGACTAGCCTGTTCA-3' |
| U6 | forward | 5'-CGCAAGGATGACACGCAAATTC-3' |
|  | reverse | 5'-GTGCAGGGTCCGAGGT-3' |
| β-actin | forward | 5'-CCTTCCTGGGCATGGAGTC-3' |
|  | reverse | 5'-TGATCTTCATTGTGCTGGGTG-3' |
| CD133 | forward | 5'-ACACTACCAAGGACAAGGCG-3' |
| OCT4  NANOG  SOX-2  Mettl3  miR-584-5p  MAPK1 | reverse  forward  reverse  forward  reverse  forward  reverse  forward  reverse  forward  reverse  forward  reverse | 5'-TCTCCAACGCCTCTTTGGTC-3'  5'-CAAAGCAGAAACCCTCGTGC-3'  5'-AACCACACTCGGACCACATC-3'  5'-GATGCCTCACACGGAGACTG-3'  5'-TTGACCGGGACCTTGTCTTC-3'  5'-AGGATAAGTACACGCTGCCC-3'  5'-TTCATGTGCGCGTAACTGTC-3'  5'-TGCTTGGTTGGTGTCAAAGG-3'  5'-AATCTTGCGAGTGCCAGGAG-3'  5'-TTATGGTTTGCCTGGGACTGAG-3'  5'-CGAATTCTAGAGCTCGAGGCAGG-3'  5'-AACAGGCTCTGGCCCACCCA-3'  5'-AGTCCTCTGAGCCCTTGTCCTGA-3' |
