# Supplementary material for: m6A Modification-Mediated DUXAP8 Regulation of Malignant Phenotype and Chemotherapy Resistance of Hepatocellular Carcinoma Through miR-584-5p/MAPK1/ERK Pathway Axis
Source: Front Cell Dev Biol. 2021 Dec 9;9:783385. doi: 10.3389/fcell.2021.783385 (PMC8696125; doi:10.3389/fcell.2021.783385)
Supplement: Supplementary file 7 [file Table2.docx]

**Table 2:** **Relationship between DUXAP8 and clinicopathological parameters in 79 HCC patients**

| Variables | All cases | DUXAP8 expression | | *P* |
| --- | --- | --- | --- | --- |
|  |  | Low (n = 40) | High (n = 39) |  |
| **Age (years)** |  |  |  | 0.214 |
| <50 | 31 | 13 | 18 |  |
| ≥50 | 48 | 27 | 21 |  |
| **Gender** |  |  |  | 0.174 |
| Male | 53 | 24 | 29 |  |
| Female | 26 | 16 | 10 |  |
| **Tumor nodule number** |  |  |  | 0.243 |
| Solitary | 56 | 26 | 30 |  |
| Multiple(≥2) | 23 | 14 | 9 |  |
| **HBV infection** |  |  |  | 0.288 |
| Positive | 63 | 30 | 33 |  |
| negative | 16 | 10 | 6 |  |
| **Tumor size (cm)** |  |  |  | **0.009** |
| <5 | 46 | 29 | 17 |  |
| ≥5 | 33 | 11 | 22 |  |
| **TNM stage** |  |  |  | **0.031** |
| Ⅰ-Ⅱ | 36 | 23 | 13 |  |
| Ⅲ-Ⅳ | 43 | 17 | 26 |  |
| **Microvascular invasion** |  |  |  | **0.001** |
| Yes | 25 | 6 | 19 |  |
| No | 54 | 34 | 20 |  |
| **AFP,μg/L** |  |  |  | 0.428 |
| <200 | 27 | 12 | 15 |  |
| ≥200 | 52 | 28 | 24 |  |
