# Supplementary material for: m6A Modification-Mediated DUXAP8 Regulation of Malignant Phenotype and Chemotherapy Resistance of Hepatocellular Carcinoma Through miR-584-5p/MAPK1/ERK Pathway Axis
Source: Front Cell Dev Biol. 2021 Dec 9;9:783385. doi: 10.3389/fcell.2021.783385 (PMC8696125; doi:10.3389/fcell.2021.783385)
Supplement: Supplementary file 9 [file DataSheet1.docx]

Supplementary Material

# Supplementary Figures

Supplementary Figure 1：Differential expression of *DUXAP8* in hepatocellular carcinoma and paraneoplastic tissues

**(A)** Comparison of the expression of *Lnc-POTEM-4*, *DUXAP8* and *Lnc-CNR1-1* in hepatocellular carcinoma tissues and paired normal paraneoplastic tissues of 15 HCC patients. **(B)** Comparison of *DUXAP8* expression in hepatocellular carcinoma tissues with normal tissues in the GEPIA database. **(C)** Correlation between *DUXAP8* expression levels and HCC prognosis in the GEPIA database.

Supplementary Figure 2: *DUXAP8* can regulate the characteristics of migration, invasion, and stemness of HCC cells

**(A)** Validation of RT-qPCR transfection effect on *shDUXAP8#1*-*shDUXAP8#6*. **(B)** RT-qPCR validating the transfection efficiency of DUXAP8 down-regulation or overexpression. **(C)** Migration and invasion. **(D)** Western Blot detection of the expression of epithelial-mesenchymal transition markers in *DUXAP8*-overexpressed HCC-LM3 cells. **(E)** Statistical analysis of primary and secondary spheroid formation capacity of *DUXAP8*-overexpressed HCC-LM3 cells, representative images showing secondary spheroid formation in these cells. **(F)** Western blot detects the expression levels of stemness-related genes in *DUXAP8*-overexpressed HCC-LM3 cells. **(G)** RT-qPCR detecting the expression of stemness-related genes in *DUXAP8*-down-regulated Huh7 and SNU-449 cells. **(H)** RT-qPCR detecting the expression of stemness-related genes in *DUXAP8*-overexpressed SK-Hep-1 and HCC-LM3 cells.

Supplementary Figure 3: *DUXAP8* promotes chemoresistance of HCC to Sorafenib

**(A)** Colony formation of HCC-LM3 cells transfected with *DUXAP8*-overexpressed plasmid after treatment with Sorafenib. **(B)** CCK8 was used to analyze the cell viability of HCC-LM3 cells transfected with *DUXAP8*- overexpressed plasmid under the effect of Sorafenib. **(C)** Effect of different concentrations of Sorafenib on HCC viability. **(D)** Whole lung specimen of nude mice after caudal vein injection of HCC cells.

Supplementary Figure 4: Effect of *DUXAP8* and miR-584-5p downregulation on the malignant phenotype of HCC cells

**(A)** RNA pull-down assay showed that miR-584-5p was highly enriched in *DUXAP8* precipitation. **(B)** Effects of *DUXAP8* down-regulation and miR-584-5p down-regulation on migration and invasion of SNU-449 cells. **(C)** Effect of down-regulation of DUXAP8 and miR-584-5p on primary and secondary spheroid formation ability in SNU-449 cells. **(D)** Effects of down-regulation of DUXAP8 and miR-584-5p on the expression levels of stemness-related genes in SNU-449 cells. **(E)** Effects of overexpression of DUXAP8 and miR-584-5p on migration and invasion of SK-Hep-1 cells. **(F)** Effects of overexpression of DUXAP8 and miR-584-5p on primary and secondary spheroid formation ability of SK-Hep-1 cells. **(G)** Effects of overexpression of DUXAP8 and miR-584-5p on the expression levels of stemness-related genes in SK-Hep-1 cells.

Supplementary Figure 5: Effect of miR-584-5p and *MAPK1* overexpression on the malignant phenotype and chemosensitivity of HCC cells.

Supplementary Figure 6: Effects of *DUXAP8* downregulation and *MAPK1* overexpression on the malignant phenotype and chemosensitivity of HCC cells.
